# Denigration of general practice as a career choice: The students’ perspective. A qualitative study

**DOI:** 10.3399/bjgpopen20X101132

**Published:** 2021-01-20

**Authors:** Emmet Carlin, Hugh Alberti, Kristen Davies

**Affiliations:** 1 Newcastle University, School of Medical Education, Newcastle, UK

**Keywords:** general practice, undergraduate education, postgraduate education, career choice, students, medical

## Abstract

**Background:**

General practice in the UK and other parts of the world is facing a recruitment crisis with insufficient numbers of medical students selecting it as a career choice. Denigration of general practice has been postulated as one of the contributing factors.

**Aim:**

To explore comments about general practice as a career made by clinical teachers from the medical student’s perspective, and in doing so to further understand the current difficulties of recruiting into general practice.

**Design & setting:**

A qualitative, explorative study of three focus groups of medical students from two medical schools in northern England.

**Method:**

A semi-structured interview format was utilised. The following four questions were posed to the participants about choosing general practice as a career: had they heard negative comments? Had they heard positive comments? Do they think comments influence student career decisions or is it a problem? Could they suggest any solutions to the issue? Results were analysed using thematic analysis.

**Results:**

Students reported hearing both positive and negative comments about general practice as a career choice. They perceived the comments to potentially influence student career choice. Three underlying themes emerged: the individual (personal characteristics of students affecting the influence that comments have on them); the curriculum (presence and content of general practice teaching); and culture (in the medical school and profession). These were used to postulate a model that may explain how negative comments shape students’ perceptions of general practice.

**Conclusion:**

Denigration of general practice is an ongoing problem within the medical profession and strategies to address it must be developed or recruitment to the specialty will continue to decline. This study suggests a model that can help to understand the complex relationship between different factors that result in negative comments being taken on board by medical students.

## How this fits in

Denigration of general practice as a career choice has been highlighted as an issue in several publications. This study confirms that medical students are exposed to the phenomenon; however, it has also found that comments made about general practice are not purely negative in nature. A model has been proposed that illustrates the interplay between the major themes of the study: the individual, the curriculum, and medical culture. The authors would suggest that this model aids the understanding of how comments that denigrate general practice shape students' perceptions of the specialty.

## Introduction

In recent years, there has been a significant increase in the primary care workload in the UK and internationally.^1^ Reasons for this include increased patient demand and the drive to move services from secondary care into the community. To address this, the Department of Health in the UK has set a target of 50% of postgraduate medical training places to be allocated to general practice.^1,2^ Consequently, the number of GP training posts has increased and have not been filled. Thus, there is a crisis in recruitment into general practice training.^3^

The literature has revealed a myriad of factors as potential influences on the career choices of students and junior doctors, such as the opinions of family and friends, the media, potential salary, and the prospect of a good work–life balance.^4–6^ Denigration of general practice as a career, or 'GP bashing', is a further issue raised in the literature.^7,8^ These issues are not restricted to the UK. Studies carried out in Australia and New Zealand have reported similar factors that influence the career choices of medical students. Indeed, they also highlight the influence of negative comments about general practice on medical student career choice.^9,10^ Focus groups and surveys of junior doctors have explored this issue and participants in these studies reported that exposure to this phenomenon started as an undergraduate.^11,12^ Work from the US suggested that students attending medical schools with higher levels of denigration produced fewer primary care clinicians,^13^ but this has not been explored in depth with medical students themselves. Therefore, a limited amount of data are available through which to understand the phenomenon of how denigration of general practice impacts on medical students and influences their careers choices. This study seeks to help address this lack of evidence.

The aim, therefore, was to explore comments about general practice as a career choice made by clinical teachers from medical students' perspectives. The study is being conducted to further understand the current difficulties in recruiting medical students into general practice.

## Method

This was a qualitative, explorative study consisting of three focus groups of year-4 medical students. Characteristics of the participants represented a cross-section of the year-4 medical student cohort at the institutions involved; that is, participants included male and female students, postgraduates and school entry, and students of various ethnic and socioeconomic backgrounds. The total number of participants was 32. The focus groups took place in two medical schools in the north of England: one newer, small institution with a high output of graduates becoming GPs; and the other a more traditional, well-established Russell group institution with a lower output of graduates becoming GPs.

Students were invited to participate by email and were sent information about the study prior to agreeing to participate. Written consent was obtained from participants prior to conducting the focus groups and further verbal consent sought after the focus groups had concluded.

A semi-structured question framework was utilised. Participants were asked about their experiences of comments made by clinical teachers about general practice as a career. Two group interviews were conducted by a GP trainee undertaking an educational integrated post and one by a final-year medical student. Data were analysed by the GP trainee. Four broad questions were posed to participants: had they heard negative comments? Had they heard positive comments? Do they think comments influence student career decisions or is it a problem? Could they suggest any solutions to the issue?

Focus groups were digitally recorded and professionally transcribed verbatim. Data were analysed using thematic analysis based on the Braun and Clarke approach.^14^

## Results

### Responses to questions and thematic analysis

In response to the questions that were posed to the participants, students reported hearing both positive and negative comments about general practice;. they perceived the comments to potentially influence career choice; and they raised some potential solutions to combat denigration.

Many examples were reported of clinical teachers making comments that denigrate general practice as a career choice (FGp = focus group 1, 2, or 3):

*'People always say, "just a GP" — like you’ve not quite made it to become a proper doctor.'* (FG p 1)

*'Please don’t tell me you are going to be a GP.'* (FGp 2)

*'This will be irrelevant for the half of you that will become GPs.'* (FGp 2)

This type of specialty 'bashing' appeared to be commonplace within the medical profession and was not solely directed towards general practice:

*'* *There seems to be this hierarchy, where right at the bottom are people to be made fun of — GPs, psychiatrists, and dermatologists* *.'* (FGp 3)

However, not all the comments that students heard from clinical teachers were negative:

*'I’ve just spent time on Geriatrics and the consultant was complimentary towards the GPs in the local area. This was due to collaborative work they’d done with the geriatricians to improve the diagnosis and treatment of heart failure in the area.'* (FGp 2)

*'I’ve had lots of positive comments from GPs themselves regarding their enjoyment of their job and advising me towards it as a career choice. Some are uncertain about the future though.'* (FGp 1)

Students stated that they perceived uncertainty about the future to be a problem given the ongoing crisis with recruitment. However, there was an understanding among the participants that the solution to the crisis was multifactorial and could not be solved by addressing the issue of denigration alone.

Some students felt that comments were unlikely to influence them personally, but they could appreciate how they might influence their peers, particularly because clinical teachers are noted to be significant role models:

*'...* [clinical teachers] *are what we see ourselves becoming and what we aspire to be.'* (FGp 1)

The participants articulated some potential solutions to the problem of denigration of general practice:

*'There’s this obvious divide between GPs and hospital. Consultants understand each other’s job more than they do with GPs, so I don’t really think they’ve much empathy. It might help if that was addressed somehow.*' (FGp 3)

*'Maybe if instead of having just a weekly placement, we had a block like the other specialties, you’d think of it as more important and you’d get a bit more of a representative view of it as a possible job.*' (FGp 2)

#### Emerging themes

**Figure 1.**
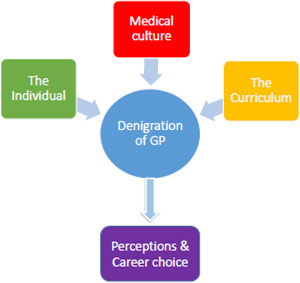
Factors that facilitate denigration of general practice to influence career choice

Three noticeable underlying themes emerged from the data analysis: the individual; the curriculum; and medical culture. Figure 1 shows how these factors could facilitate denigration of general practice and how they interplay to influence career choice.

##### The individual

This refers to the type of individual hearing the denigratory comment. Participants felt that certain personal characteristics would mitigate the impact of denigratory comments about general practice (or other specialties). The following characteristics were suggested that would make students more likely to be influenced by the comments: being a school-leaver entrant student compared with a graduate-entry student; being in the early years of the degree compared with being in the later years; and being unclear about the career one wishes to pursue.

*'I think it depends when you hear comments … in first and second year you are quite impressionable.*' (FGp 1)

*'I feel like if I was younger and come in on the five-year course then they would influence me a lot more.'* (FGp 3)

*'Negative comments definitely have some impact … that it’s an easy option or you’re less intelligent if you go into GP… it doesn’t put me off, but I can see how it might put someone else off.*' (FGp 1)

##### The curriculum

This theme relates to the presence that general practice is afforded on the undergraduate curriculum. Participants referred to the topics that GPs are taught within the curriculum and the perception of these as 'soft' compared with basic science, or certain or specific clinical specialties. Furthermore, students felt that there was not sufficient clinical time spent in general practice when compared with secondary care, which they believed implied that it was of less importance. The little attention paid to general practice by the formal curriculum reinforced the negative attitudes towards the specialty, which are already being experienced by students as part of the professional or medical school culture.

*'In our first and second year we get very poor teaching about GP… in third year you only go on a Wednesday morning and leave without getting an understanding of how it* [general practice] *works.'* (FGp 2)

*'Part of the reason I feel negative towards GP is the standard of teaching … the lectures based on GP were on health beliefs … they were the driest, dullest things … I can see why people perceive it as being a specialty for the less intelligent … I think it’s how it is taught at medical school.'* (FGp 1)

*'If its presence on the curriculum had as big an emphasis as other specialties, I think you’d be more inclined to take* [negative] *comments on the chin.'* (FGp 3)

##### Medical culture

This theme refers to the implicit or prevailing culture both within medical schools and the profession as a whole. Some students reported feeling apprehensive about voicing their desire to pursue a career in general practice for fear of the response from colleagues and clinical teachers. General practice is perceived to be a less challenging and less competitive career choice than other specialties, and is seen to appeal to for those who want an easier life:

*'There is a sense that GP is for those who want part-time work … this is perceived as a negative thing … that you’re out for an easy life.'* (FGp 2)

*'It’s easier to get into, you have to compete a lot harder to get into surgery and medicine … it’s seen as less competitive and therefore a compromise.*' (FGp 2)

*'It’s just like a whole culture where it’s not taken seriously … I feel like people are ashamed to vocalise it as their career plan.'* (FGp 3)

## Discussion

### Summary

The study confirms that undergraduate students experience comments that denigrate general practice as a career choice from their clinical teachers. This is consistent with other work undertaken to date.^11,14^ Participants agreed that this issue can potentially influence the career choices of their peers.

The findings suggest that there is a complex interplay between three underlying factors: the individual, the curriculum, and medical culture (see Figure 1). These factors may help to explain how denigratory comments are taken on board by some medical students and subsequently shape their perceptions of general practice as a prospective career choice.

### Comparison with existing literature

Denigration of general practice has been highlighted in several previous publications.^8,11,15^ This study further confirms this as a factor that may deter students from pursuing a career in general practice. The themes within the data of this study have been alluded to in other work also, albeit under different titles.^15,16^ The theme of ‘the curriculum’ has been referred to in previous relevant work as the ‘formal curriculum’.^8,17^ The theme of the individual is relatively novel, and it is postulated that personal characteristics of students may indeed determine the likelihood of denigratory comments influencing their career choice.

The theme of medical culture could be considered to be the ‘hidden curriculum’, which has been highlighted in other studies of student perceptions of general practice.^17^ Medicine is by its nature and entry requirement a competitive career choice and environment. Therefore, when comments are made that allude to the fact that general practice is not challenging, or is a ‘good fallback’ career option, this will clearly have a negative influence on students' perceptions of the specialty as a career choice. Furthermore, this theme can be considered through the lens of various theoretical frameworks, such as social theory and sociocultural learning. It is an issue that should be analysed and addressed in a similar manner to other discriminatory practices in medicine, such as the lack of representation of women in academic medicine.^18^

### Strengths and limitations

This study was carried out at two medical schools in the UK in order to enhance its transferability to other similar contexts, but the relatively small sample size is acknowledged. Standard means to ensure rigour in qualitative research were followed.^19^ It is believed the description offered of the context in which this research was conducted will add to the transferability of its findings. Reference has also been offered to the precise method of thematic analysis used, along with clear explanations of the data collection methods. As such, it is believed this adds to the dependability of the study. With respect to confirmability, the primary researcher acknowledges he has approached the research from the perspective of a young GP with an enthusiasm for his chosen career and a desire to encourage colleagues into the specialty. Consequently, he has striven to minimise the influence this may have on the conduct of the study.

Participants may have had a positive perception of general practice given that they agreed to participate in the study; although, this was not formally asked. Owing to its retrospective nature, participants may also have been prone to recall bias.

A further limitation of this study was the fact all participants were at the same stage of their medical school training. Collecting data from students in other year groups and doctors in the foundation programme may provide a deeper insight into the phenomenon being studied.

### Implications for practice

By understanding the underlying factors that provide the basis for denigratory comments to take root and shape medical students' perceptions of general practice, more robust steps can be taken to address the problem. The issues raised by the participants in this study could be specifically addressed in the following ways, some of which were specific recommendations in the Health Education England and Medcial Schools Council report *B* *y choice* *—* *not by chance:* ^8^ (1) change university or medical school policy to a zero tolerance approach towards denigration of any specialty. This may not be possible in clinical environments but certainly should be attainable within the university itself and among employees; (2) publicise and deliver education within the medical profession about the impact that denigratory comments can have on students. Colleagues and clinical teachers should be aware of their position as role models; (3) parity of esteem or increased exposure to general practice on the undergraduate curriculum. Furthermore, GPs should be provided with opportunities to teach more clinical topics within curricula; (4) enhanced understanding between primary and secondary care. A better appreciation for each other’s roles between primary and secondary care clinicians would foster mutual respect, and address workforce issues at a fundamental level.

This study sought to explore medical students' experiences of denigration of general practice by their clinical teachers. The study has confirmed that medical students are exposed to the phenomenon, but it has also found that comments made about general practice are not purely negative in nature. A model is proposed that illustrates the interplay between the major themes of the study: the individual, the curriculum, and medical culture. It is suggested that this model aids the understanding of how comments that denigrate general practice shape students' perceptions of the specialty. It is believed that the suggested recommendations for practice, alongside other measures, will go some way to help address the current difficulties in recruitment to general practice.
